# Intravenous immunoglobulin G in the treatment of ABO hemolytic disease of the newborn during the early neonatal period at a tertiary academic hospital: a retrospective study

**DOI:** 10.1038/s41372-021-00963-5

**Published:** 2021-02-15

**Authors:** Jiarong Pan, Canyang Zhan, Tianming Yuan, Xiangxiang Chen, Yanyan Ni, Ying Shen, Weiwei Chen, Tai Wu, Huimin Yu

**Affiliations:** Department of Neonatology, the Children’s Hospital, Zhejiang University School of Medicine, National Clinical Research Center for Child Health, Hangzhou, Zhejiang People’s Republic of China

**Keywords:** Immunological disorders, Paediatrics, Outcomes research

## Abstract

**Objective:**

To evaluate the efficacy and safety of intravenous immunoglobulin G (IVIG) in infants with ABO hemolytic disease of the newborn (HDN).

**Methods:**

Infants with moderate-to-severe ABO HDN during early neonatal period (<7 days) at our hospital in 2017 were included in this retrospective study. Patients treated with IVIG and phototherapy were classified as the IVIG group, and those who only received phototherapy were classified as the phototherapy only group.

**Results:**

Forty-six patients were classified into the IVIG group and 68 other patients were classified into the phototherapy only group. There was no significant difference in duration of phototherapy, hospitalization periods, needs for exchange transfusion, transfusions, and incidence of bilirubin-induced neurological sequelae between these two groups (*P* = 0.20, 0.27, 0.65, 0.47, 0.78, respectively).

**Conclusion:**

It seems unnecessary to expose neonates to IVIG in moderate-to-severe ABO HDN when the available data show no appreciable benefits.

## Introduction

ABO hemolytic disease of the newborn (HDN) is the most common alloimmune hemolytic disease in neonates. Approximately 25% of all maternal/fetal pairs are ABO-incompatible, while ABO HDN occurs in less than 1% of such group O women with antenatal high-titer IgG antibodies [1]. In the postnatal period, infants may exhibit hyperbilirubinemia and anemia, which results in considerable neonatal morbidity and mortality. There may be some complications including bilirubin-induced neurologic dysfunctions if the infants do not receive appropriate treatment [2]. The conventional treatment for ABO HDN consists of phototherapy and exchange transfusion [3]. Because exchange transfusion is an invasive procedure with high risk, alternative treatments such as intravenous immunoglobulin G (IVIG) have been applied in infants diagnosed with ABO HDN [4]. Despite IVIG is thought to reduce the rate of hemolysis and consequently the need for exchange transfusions in theory, the conclusions of the previous research on the use of IVIG in ABO HDN are controversial in recent years [5]. Furthermore, as a kind of blood product, the use of IVIG has been associated with significant morbidity in neonates [6, 7]. Therefore, additional clinical research evaluating IVIG in ABO HDN need to be performed before definitive conclusions can be drawn.

We aim to establish appropriate indications for IVIG use in ABO HDN by evaluating the effect of IVIG in neonates with moderate-to-severe ABO HDN during the early neonatal period (<7 days).

## Patients and methods

This was a single-center retrospective study of IVIG use in neonates with ABO HDN at the Children’s Hospital, Zhejiang University School of Medicine from January 1 to December 31 in 2017. Inclusion criteria included hospitalized neonates in the department of neonatology for treatment of ABO HDN as determined by the treating clinicians, availability of bilirubin levels, positive direct antiglobulin test (DAT) and/or positive antibody on infant’s red blood cells (RBCs). The DAT and antibody release tests were performed by gel microcolumn assay which is a sensitive method to detect RBC alloantibodies. The diagnosis of ABO HDN was confirmed by positive DAT and/or positive antibody release test indicating antibodies on RBCs in our study. The infants with ABO HDN who met the total serum bilirubin level inclusion criteria (within 3 mg/dL (51 µmol/L) of exchange transfusion level or even above exchange transfusion level) (Table 1) were included in the study according to the guidelines from the American Academy of Pediatrics (AAP) [3].

**Table 1:** Total serum bilirubin levels of the inclusion criteria.

Postnatal day	Total serum bilirubin levels (μmol/l)	
	GA 35–38 weeks	GA ≥ 38 weeks
1st	≥205	≥230
2nd	≥240	≥275
3th	≥265	≥315
4–7th	≥275	≥335

*GA* gestational age.

Exclusion criteria include lack of bilirubin level recordings within 1 day of IVIG administration (pre or post), IVIG given after the age of 7 days, IVIG given for a reason other than ABO HDN, and associated risk factors that could increase bilirubin level such as infection, prematurity (less than 35 weeks of gestational age), G6PD deficiency, polycythemia, visceral hemorrhage, and cephalohematoma. In all cases, gender, gestational age, birth weight, and the postnatal age at which the diagnosis was made and the treatment were recorded. Informed consent was obtained from all parents or guardians. All of the clinical data included were fully anonymized and can no longer be retraced. The study protocol was approved by the ethics committee of the Children’s Hospital, Zhejiang University School of Medicine.

Patients who fulfilled the above criteria were included and classified into two groups: The IVIG group received IVIG and light-emitting diodes (LED) phototherapy whereas the phototherapy only group received only LED phototherapy alone. In this study, we retrospectively collected data from the IVIG group and the phototherapy only group. The outcomes of interest used to evaluate the efficacy and safety of IVIG in infants with ABO HDN were as follows: duration of phototherapy, hospitalization periods, needs for exchange transfusion, transfusions, and incidence of bilirubin-induced neurological sequelae.

All quantitative values were expressed as arithmetic mean ± standard deviation (SD). The quantitative and nominal data analyses were conducted using independent sample *t*-tests and Chi-square tests respectively. All analyses were performed using SPSS Statistics for Windows (software version 26.0).

## Results

**Fig. 1:**
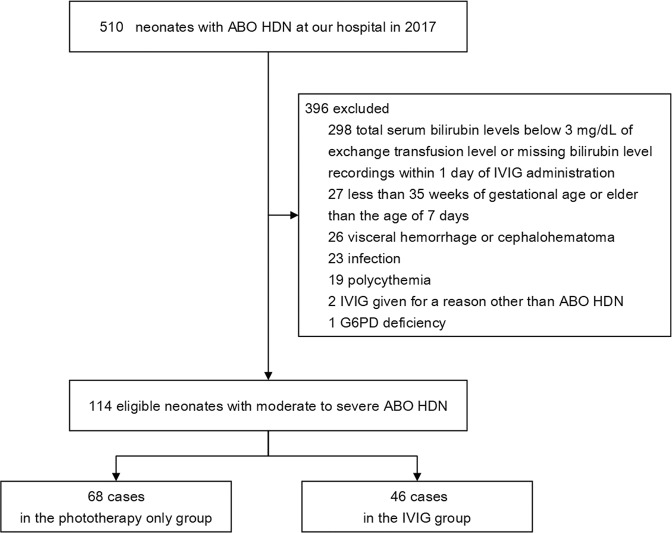
Study Period and Population. Study profile: a total of 510 cases of neonates were diagnosed with ABO HDN at our hospital in 2017, and 114 of them were included in the study with 68 cases in the phototherapy only group and 46 cases in the IVIG group.

A total of 510 cases of neonates were diagnosed with ABO HDN at our hospital in 2017, and 114 of them were included in the study according to inclusion and exclusion criteria (Fig. 1). Median age was 2.85 ± 1.73 (arithmetic mean ± SD) days (range 0–7 days). Median gestational age was 38.34 ± 1.44 (arithmetic mean ± SD) weeks (range 35–41 weeks). There were 68 cases in the phototherapy only group and 46 cases in the IVIG group. Demographic and clinical characteristics of the study cohort are summarized in Table 2. All patients in the two groups were fed orally with breast milk or formula milk. The volumes of oral intake were adjusted according to their clinical status and body weight changes associated with treatment during hospitalization. Complete blood, reticulocyte and differential counts, hematocrit, total bilirubin, DAT, hepatic and renal function, blood cultures, and C-reactive protein determinations were performed routinely in all patients. There were no significant differences between the two groups with respect to gender, gestational age, birth weight, postnatal age, bilirubin level, hematocrit level, hemoglobin level, or reticulocyte count (*P* = 0.542, 0.65, 0.64, 0.37, 0.06, 0.36, 0.22, 0.06, respectively).

**Table 2:** Demographic and clinical characteristics of the newborn patients.

	Phototherapy only group	IVIG group	*P*
	(*n* = 68)	(*n* = 46)	
Gender (male/female)	33/35	21/25	0.542
Gestational age (weeks)	38.29 ± 1.61	38.41 ± 1.15	0.65
Birth weight (g)	3248.75 ± 412.18	3285.93 ± 408.51	0.64
Postnatal age (days)	2.97 ± 1.83	2.67 ± 1.56	0.37
Bilirubin level (μmol/l)	335.72 ± 74.64	364.24 ± 86.18	0.06
Hematocrit level (%)	47.94 ± 6.72	46.68 ± 7.69	0.36
Hemoglobin level (g/l)	153.78 ± 19.83	148.74 ± 23.29	0.22
Reticulocyte count (%)	4.50 ± 2.85	6.02 ± 4.72	0.06

The included newborns in the IVIG group received IVIG (Boya biopharmaceutical Co., Ltd, Jiangxi, China) at a single dose of 0.5–1 g/kg for a 2 h intravenous infusion as soon as the diagnosis of ABO HDN was confirmed. They were closely monitored for possible febrile, allergic, symptoms and signs of necrotizing enterocolitis and volume overloading side effects of IVIG therapy, including changes in respiratory rate and pattern, heart rate, blood pressure, skin color, and abdominal tension. No adverse effects were observed during IVIG treatments.

**Fig. 2:**
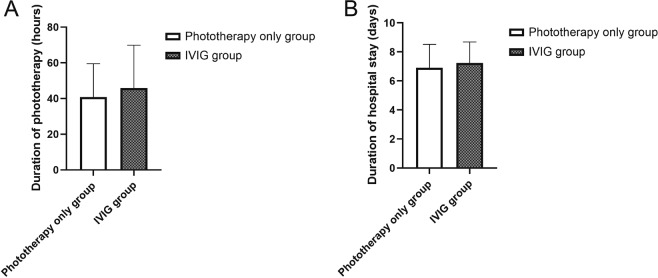
Effect of IVIG on Duration of Phototherapy and Hospital Stay. Duration of phototherapy and hospital stay: **A** duration of phototherapy in the two groups. **B** duration of hospital stay in the two groups. The bars represent arithmetic means and the error bars represent standard deviations. There was no significant difference between the two groups with respect to the duration of phototherapy and hospitalization periods (*P* = 0.20, 0.27, respectively).

Every newborn patient received LED phototherapy treatment after admission to the neonatal department and received repeated phototherapy treatment when a total serum bilirubin concentration was higher than phototherapy level of guidelines from the AAP again, regardless of IVIG therapy. Phototherapy was provided with LED phototherapy system (Ningbo David Medical Device Co., Ltd, Zhejiang, China, intensity ≈ 30 μW/cm^2^/nm, spectrum 450–480 nm). And phototherapy treatment was discontinued as soon as the bilirubin level decreased to a safe threshold according to the guidelines from AAP. There was no significant difference between the two groups with respect to the duration of phototherapy and the hospitalization periods (*P* = 0.20, 0.27, respectively) (Fig. 2).

**Fig. 3:**
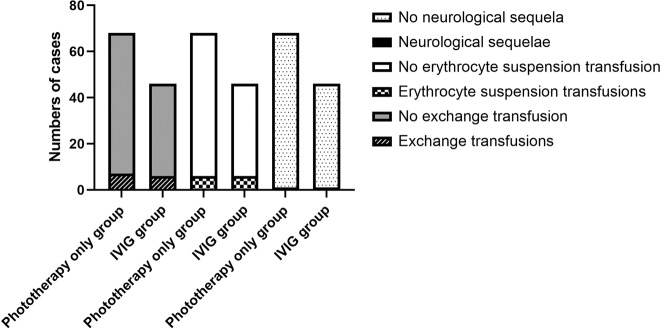
No Advantages of IVIG in Avoiding Exchange Transfusions, Erythrocyte Suspension Transfusions or Neurological Sequelae. Comparisons of numbers of cases treated with exchange transfusion, transfusions, and cases complicated with bilirubin-induced neurologic sequelae between these two groups: there was no significant difference in needs for exchange transfusion, transfusions, and incidence of bilirubin-induced neurological sequelae between these two groups (*P* = 0.65, 0.47, 0.78, respectively).

Exchange transfusions were performed when total serum bilirubin concentrations met or exceeded AAP exchange transfusion thresholds despite both treatment maneuvers. There were 13 patients that had met or exceeded AAP exchange transfusion thresholds and received exchange transfusions. Seven cases were in the phototherapy only group and other six cases were in the IVIG group. There was no significant difference between the two groups with respect to exchange transfusions (*P* = 0.65) (Fig. 3). Three of the patients who had exchange transfusion in the phototherapy only group developed acidosis, hypotension, and hypocalcaemia after exchange transfusions during the initial hospitalization. These complications were all treated promptly and appropriately.

There were 12 patients who received packed erythrocyte suspension transfusions when they had symptoms and signs of anemia with hemoglobin levels significantly decreased below the normal range. Of the 12 patients with transfusions, six cases were in the phototherapy only group and other 6 cases were in the IVIG group. There was no significant difference between the two groups with respect to transfusions (*P* = 0.47) (Fig. 3).

The patients who received exchange transfusion were either followed up through an appointment to attend the outpatient clinic or by telephone from December 2017 to May 2020. Twelve of the 13 patients who received exchange transfusions were successfully followed up till May 2020 except that one patient died from accident in 2019. Magnetic resonance imaging (MRI) of brain and brainstem auditory evoked potential for neonates were performed for them. Among the 12 cases of successful follow-up, ten patients had no signs of disease or abnormal development, but the other two had significant bilirubin-induced neurological sequelae. One case had acute bilirubin encephalopathy on admission, and eventually all resolved except hearing impairment in the phototherapy only group, and the other case whose peak total serum bilirubin level was over 600 µmol/L had acute bilirubin encephalopathy and eventually developed typical icteric encephalopathy in the IVIG group. There was no significant difference between the two groups with respect to the incidence of neurological sequelae (*P* = 0.78) (Fig. 3). T1-weighted brain MRI of the neonate who eventually developed typical kernicterus showed increased signal in the basal ganglia bilaterally. Currently age 3, this child is seriously disabled with mental retardation, dystonia and athetosis that he needs special care.

## Discussion

Newborns with ABO HDN are at high risk for hyperbilirubinemia due to alloimmune hemolysis. In cases of ABO incompatibility, maternal immunoglobulin G antibodies of anti-A or anti-B pass through the placenta and attach to the specific sites of antigen A or antigen B on the fetal or neonatal erythrocytes [8]. Infants with ABO HDN usually encounter hyperbilirubinemia when heme catabolism increases excessive bilirubin production due to destructions of antibody-coated erythrocytes by immune cells of the reticuloendothelial system. All newborns included in our research were diagnosed as having moderate-to-severe hyperbilirubinemia during the early neonatal period due to ABO HDN confirmed by a positive DAT and/or positive antibodies on infants’ RBCs. It was found that IVIG had therapeutic effects on the reduction of maternal antibody titers and severity of fetal hemolysis in pregnant women with Rh hemolytic diseases by Rewald and Suringar in 1965 [9]. Subsequent case reports and clinical research reported IVIG had therapeutic effectiveness in the management of neonates with both Rh and ABO incompatibility [10–12]. IVIG is a pooled medication of human immunoglobulins obtained from several thousand healthy donors and being widely used to treat a variety of diseases at present [13]. IVIG consists of proteins (mainly IgG antibodies, as well as IgM, IgA, and IgE antibodies, albumin, cytokines), sugar, electrolytes, and solvents [14]. The possible therapeutic mechanisms of IVIG in neonates with ABO HDN include complicated biological processes of suppressing the phagocytosis of antibody-coated RBCs by Fc blockade, neutralizing antibodies, and therefore the rate of alloimmune hemolysis is reduced and hyperbilirubinemia induced toxicity may be avoided. However, some clinical research found that IVIG treatment failed in the management of many cases with ABO HDN. It remains a controversy whether use of IVIG in ABO HDN should be recommended now [15]. Therefore, additional clinical studies are warranted to establish appropriate indications for IVIG use in the treatment of ABO HDN.

This study focuses the demographic and clinical characteristics, the needs for exchange transfusions, the durations of phototherapy, hospitalization periods, and incidence of bilirubin-induced neurological sequelae in a cohort of neonates with ABO HDN who received treatment with IVIG. According to the demographic and clinical characteristics, the initial conditions of the two groups including the severity of hemolysis are similar. The age of the included cases is in the early neonatal period during which the rate of alloimmune hemolysis is rapid and newborns are at high risk for a potentially devastating condition of acute bilirubin encephalopathy that can result in death or life-long neurodevelopmental handicaps.

Phototherapy is a kind of proven and standard treatment for most neonates with hyperbilirubinemia, which could help avoiding bilirubin-induced neurological dysfunctions including acute bilirubin encephalopathy and kernicterus, but it is recommended to use phototherapy for the shortest possible time because of potential side effects of phototherapy for newborns [16, 17]. In our study, there were no significant differences in the duration of phototherapy and hospitalization periods between the two groups. These results revealed that IVIG therapy has no effectiveness in shortening the duration of phototherapy and hospital stay in patients with moderate-to-severe ABO HDN during the early neonatal period. It is speculated that IVIG cannot block the hemolysis process or change the rate of bilirubin production after the RBCs being sensitized by antibodies.

Exchange transfusion is an effective treatment for neonates with severe hyperbilirubinemia [18, 19]. Exchange transfusion that could promptly reduce bilirubin levels involves removal of the newborn’s blood with high levels of circulating bilirubin and simultaneous replacement with compatible donor blood. Exchange transfusion could not only reduce bilirubin levels but also remove a lot of sensitized RBCs and circulating antibodies. However, as exchange transfusion is an invasive procedure, there are many potential complications including cardiac and respiratory concerns, catheter-related complications, infection, hypothermia, hypoglycemia, thrombosis, electrolyte imbalance (e.g., hypocalcemia), acidosis, and necrotizing enterocolitis [20, 21]. The neonates that had severe ABO HDN and met or exceeded exchange transfusion criteria received exchange transfusion therapy in our study, and there was no significant difference in the need for exchange transfusion between the two groups. It showed that IVIG administration has no advantages over avoiding exchange transfusion which is a risky and invasive procedure for newborns.

Most of anemic newborns due to ABO HDN may receive routine care. Routine care means to ensure adequate nutritional support and closely monitoring of hemoglobin concentration, bilirubin level and reticulocyte count. Type O washed RBC transfusions are only required in those with very low hemoglobin concentration or symptomatic anemia. In the present study, there was no significant difference between the two groups with respect to transfusions, and therefore IVIG therapy seems to have no advantages in avoiding transfusions.

In conclusion, although IVIG is being seen as an appropriate and effective medication in the treatment of neonates with ABO HDN for a long time, our study has shown that IVIG therapy has no advantages in this disease and it seems unnecessary to expose neonates to IVIG in moderate-to-severe ABO HDN during the early neonatal period. There are a few limitations in our study. First, this study is a single-center retrospective clinical study with a relatively small sample. Second, the study has not observed different dosages of IVIG therapy in neonates with ABO HDN. Third, there is a significant lack of data on the long-term outcomes of the infants with ABO HDN who have received IVIG therapy during the early neonatal period. The effects of IVIG therapy should be further studied in additional studies that could focus on larger numbers, different IVIG dosage, and long-term neurological outcomes of neonates with ABO HDN.
